# Extracellular vesicles as vehicles for small non-coding RNA therapeutics: standardization challenges for clinical translation

**DOI:** 10.20517/evcna.2025.33

**Published:** 2025-08-01

**Authors:** Lucrezia Luisotti, Lorenzo Germelli, Rebecca Piccarducci, Chiara Giacomelli, Laura Marchetti, Claudia Martini

**Affiliations:** ^1^Department of Pharmacy, University of Pisa, Pisa 56126, Italy.; ^2^Center for Instrument Sharing, University of Pisa, Pisa 56126, Italy.

**Keywords:** Extracellular vesicles (EVs), EV engineering, small non-coding RNA, RNA therapy, drug delivery, therapeutic dosing, standardization challenges

## Abstract

**Aim:** Extracellular vesicles (EVs) have emerged as promising vehicles for the delivery of small non-coding RNAs (sncRNAs); however, their clinical translation is hindered by the lack of standardized manufacturing methods, RNA loading protocols, and dosing strategies in both preclinical and clinical settings. This review aims to analyze the current landscape of EV-based RNA therapeutics to identify key trends and discrepancies, providing insight for the clinical development of future sncRNA-loaded EVs.

**Methods:** PubMed and Google Scholar were used to identify 74 published articles using cell-derived EVs loaded with sncRNA. EV source, EV surface modifications, type of loaded RNA, loading methods, and dosages used in preclinical studies were quantitatively analyzed to identify trends and discrepancies.

**Results:** Most studies utilize naïve EVs derived from stem or immortalized cells, with electroporation and donor cell transfection being the predominant RNA loading strategies. EV loading and dosage schemes in preclinical studies are mainly based on protein content, while only a minority of studies use particle number. More generally, the variability in measurement units reflects the absence of standardized guidelines for both RNA loading and treatment dosing, generating variability and challenges in comparing results across studies.

**Conclusion:** Reliable dosing strategies are extremely important for determining the therapeutic potential of EVs in preclinical settings and ensuring clinical translatability. However, a standardized framework for EVs as robust platforms for RNA delivery remains to be established. We underscore the critical need for universal quantification methods, standardized measurement units, and reproducible protocols for EV production and application.

## INTRODUCTION

RNA therapeutics comprise a heterogeneous class of RNA molecules that modulate biological pathways and functions by regulating the expression or activity of target molecules. This extraordinary ability to target highly different biological components, spanning from messenger RNAs to proteins, has revolutionized therapy development, offering groundbreaking approaches for disease prevention and treatment, enabling precision medicine and treatments for rare diseases^[1,2]^. Among RNA-based therapies, small non-coding RNAs (sncRNAs), including small interfering RNAs (siRNAs), microRNAs (miRNAs), and antisense oligonucleotides (ASOs), have gained particular attention for their potential to regulate gene expression and modulate disease pathways. siRNAs and miRNAs are both double-stranded (ds) RNA and function through the RNA interference (RNAi) pathway^[3]^. In this mechanism, dsRNAs are loaded into the RNA-induced silencing complex (RISC) in the cytoplasm, guiding the sequence-specific degradation of targeted messenger RNAs (mRNAs) and/or inhibition of protein expression. Once incorporated into RISC, the dsRNA is unwound into single strands. For siRNA, the sense strand is degraded, while the antisense (guide) strand remains bound to RISC. Then, the guide strand directs RISC to a fully complementary target mRNA sequence, resulting in precise mRNA cleavage and gene silencing. For miRNA, the guide strand is similarly retained, while the passenger strand is degraded. The mature single-stranded miRNA directs RISC to target mRNAs, typically binding to partially complementary sequences within the 3’ untranslated region (3’UTR), leading to either mRNA cleavage or translational repression. siRNA-based therapeutics involve the delivery of synthetic siRNAs into target cells to initiate RNAi, resulting in specific degradation of the target mRNA and gene silencing^[4]^. miRNA-based therapeutics are categorized into two main strategies: miRNA mimics and miRNA inhibitors^[5]^. miRNA mimics are synthetic, double-stranded RNAs designed to restore or enhance the function of endogenous miRNAs, while miRNA inhibitors are chemically modified, single-stranded oligonucleotides that block endogenous miRNAs by sequence complementarity. The two main categories of miRNA inhibitors are anti-miRNA oligonucleotides (AMOs) and miRNA sponges^[6]^. Anti-miRNA oligonucleotides (AMOs), originally called antagomirs^[7]^, specifically bind to endogenous miRNA and abolish its activity by inhibiting the binding of the RISC-loaded miRNA guide strand to target mRNAs^[8]^. miRNA sponges are plasmid-encoded antisense sequences that competitively bind to their target miRNAs, preventing their hybridization onto target mRNAs^[9]^. Unlike AMOs, which transiently inhibit miRNA, miRNA sponges are expressed from transgenes transfected into cells, enabling long-term miRNA inhibition^[5]^. ASOs represent another RNA therapeutic strategy. They are short, single-stranded (ss) oligonucleotides with RNA- or DNA-based structures, designed to bind complementary messenger RNA (mRNA) sequences through Watson-Crick base pairing, thereby modulating mRNA function and related protein expression^[10]^.

Despite their potential, the successful clinical translation of RNA drugs remains largely hindered by challenges in delivery. Naked RNA molecules are inherently unstable, hydrophilic, and negatively charged, making them highly susceptible to rapid degradation by ubiquitous RNases and significantly limiting their cellular uptake. These physicochemical properties also prevent RNA from passively crossing lipid bilayers. Furthermore, exogenous RNA often triggers strong immune responses, leading to potential cell toxicity^[2,11]^.

Hence, the development of efficient delivery systems to protect RNA from the harmful physiological environment is crucial to the safe and efficient delivery of RNA-based therapeutics.

Extracellular vesicles (EVs) are heterogeneous lipid bilayer vesicles released by all cell types^[12,13]^. EVs can be divided into three main subtypes according to their size and biogenesis pathway: apoptotic bodies (~500-5,000 nm), released by bubbling of the plasma membrane during cell death; microvesicles (~100-1,000 nm), directly shed by plasma membrane outward budding; and exosomes (~30-150 nm), originated via the endocytic endosomal pathway^[14]^. Exosomes play a crucial role in intracellular communication by transporting biomolecules such as proteins, lipids, and nucleic acids between cells. This intrinsic ability to transfer molecular cargoes, along with their unique structure and biological origin, makes exosomes appealing vehicles for RNA delivery^[15,16]^. Their lipid bilayer structure enables the efficient encapsulation and protection of RNA from enzymatic degradation, as well as ensuring permeation through biological barriers, including the blood-brain barrier. As natural nanoparticles, exosomes exhibit high biocompatibility and low immunogenicity, minimizing the risk of adverse immune responses. Additionally, exosomes can be engineered to target specific cells or tissues, enhancing the precision of RNA delivery^[17,18]^.

Despite extensive research over the past decade on the development of exosome-based RNA therapeutics, spanning *in vitro* models, preclinical studies, and clinical applications, no exosome-based RNA therapy has yet received approval from either the Food and Drug Administration (FDA) or the European Medicines Agency (EMA)^[19,20]^.

The manufacturing and characterization of EVs, along with their loading with therapeutic RNA, need to be standardized to improve their clinical translatability. To date, therapeutic EV research is hindered by inconsistencies in EV isolation, quantification, and EV-to-RNA ratio, as well as accurate and reliable *in vivo* dosing, limiting clinical translation^[21]^.

Within this conceptual framework, this review aims to provide a comprehensive, critical, and quantitative systematic analysis of mostly preclinical studies on sncRNA-loaded EVs, with a particular emphasis on variability in loading and dosing strategies, an aspect that has remained largely underexplored in the current literature. First, we present an overview of the key aspects in EV development as delivery systems, including the used cell sources, surface modifications for targeted delivery, RNA cargo types, and therapeutic outcomes. Next, we compare loading methods and protocols, with a particular focus on the quantification of loaded sncRNA. Finally, we assess EV dosage *in vitro*, examining the amount of sncRNA-loaded EVs delivered to cells, and *in vivo*, comparing single *vs.* multiple administrations, targeted disease, and routes of administration. Crucially, this review offers a data-driven, quantitative comparison of current procedures in the field. By identifying key trends, as well as discrepancies or inconsistencies, we highlight the urgent need for standardized quantification methods, reproducible production protocols, and universal measurement units to facilitate the clinical translation of EV-based RNA therapeutics.

## METHODS

### Articles search and inclusion criteria

**Figure 1:**
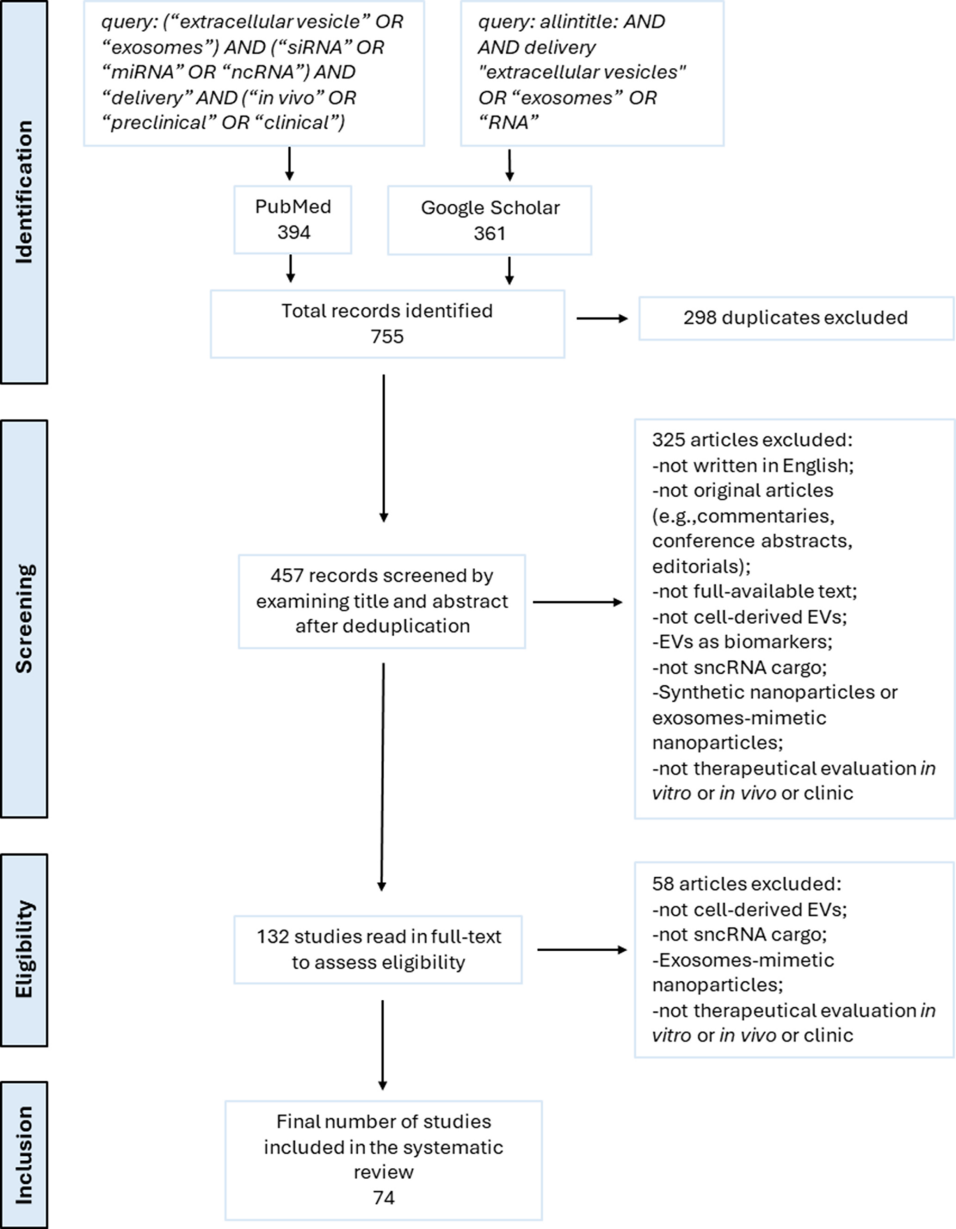
PRISMA flow diagram illustrating the study screening and selection procedures. The literature search was conducted in PubMed and Google Scholar, limited to studies published between January 2013 and January 2025. A total of 74 studies met all inclusion criteria and were included in the final analysis.

To carry out a systematic review of preclinical and clinical studies on sncRNA-loaded EVs, we performed a literature search using PubMed and Google Scholar as the main databases. The search strategy for PubMed included the following query: (“extracellular vesicle” or “exosomes”) and (“siRNA” or “miRNA” or “ncRNA”) and “delivery” and (“*in vivo*” or “preclinical” or “clinical”). For Google Scholar, the query was: allintitle: and and delivery “EVs” or “exosomes” or “RNA”. The search was limited to studies published between January 2013 and January 2025. A total of 755 records were identified (394 from PubMed, 361 from Google Scholar). After importing into Zotero, 298 duplicates were removed. The remaining 457 records were screened by title and abstract to identify studies involving cell-derived EVs loaded with small non-coding RNAs, which evaluated therapeutic delivery *in vitro* and/or *in vivo* within a preclinical or clinical framework. Studies were excluded if they were non-original publication types (e.g., commentaries, conference abstracts, editorials), not available in full text, written in languages other than English, employed synthetic nanoparticles or exosome-mimetic nanovesicles, used EVs as diagnostic or prognostic biomarkers, utilized EVs not derived from cultured cells (e.g., those from milk, bacterial, or plant sources), or used EVs that were either unloaded or loaded with other cargo types such as mRNA, proteins, or chemical drugs. After abstract screening, 132 articles were read in full to determine satisfaction with the same eligibility criteria as above. As a result, 74 studies were finally included in the systematic review. The selection process is illustrated in the PRISMA flow diagram [Figure 1].

### Categorization of selected studies per cellular EV source

To categorize the selected studies based on the EV source, we divided the EV donor cell cultures into four subgroups: stem and progenitor cells, immortalized cells, cancer cells, and primary cells. “Stem and progenitor cells” group includes: mesenchymal stem cells (MSCs), adipose-derived stem cells (ADSCs), adipose-derived MSCs (ADMSCs), bone marrow-derived MSCs (BMSCs), human adipose stem cells (hASCs), human umbilical cord MSCs (hUCMSCs), human umbilical cord blood mononuclear cells (hUCB-MNCs), cord blood mesenchymal stem cells (cbMSCs), induced pluripotent stem cells (iPSCs), induced neural stem cells (iNSCs), and endothelial progenitor cells (EPCs). “Immortalized cells” group includes: human embryonic kidney cells (HEK293, HEK293T, Expi293F), mouse embryonic fibroblasts (MEFs), human neuroblastoma cells (SH-SY5Y), murine macrophage cells (RAW264.7), murine brain endothelial cells (bEND.3), and human fibroblast cells (BJ). “Cancer cells” include: A549 (lung adenocarcinoma), HepG2 (hepatocellular carcinoma), PANC-1 (pancreatic carcinoma), SW1116 (colorectal adenocarcinoma), MDA-MB-231 (triple-negative breast cancer), A375 (human melanoma), SKMel-5 (melanoma), SKMel-28 (melanoma), MG63 (osteosarcoma), and OCI-MY5 cells (human myeloma cell line). “Primary cells” group includes: dendritic cells (DCs) and human umbilical vein endothelial cells (HUVECs).

### Categorization of selected studies per targeted disease

For *in vivo* analysis of EV dosage expressed as µg per animal, we divided publications into four groups: neurodegenerative and neurological diseases, cardiovascular disease, cancer, and others. “Neurodegenerative and neurological diseases” group includes Parkinson’s disease, Alzheimer’s Disease, Cocaine-mediated microglial activation, and Spinal cord injury. “Cardiovascular diseases” group includes: myocardial damage, myocardial infarction, and Myocardial ischemia-reperfusion injury. “Cancer” group includes: Breast cancer, Lung cancer, Hepatocellular carcinoma, Pancreatic cancer (including pancreatic ductal adenocarcinoma), Gastric cancer, Prostate cancer, Esophageal cancer, Colorectal carcinoma, Colorectal cancer, Glioblastoma, Osteosarcoma, Bladder cancer, Melanoma, Multiple myeloma, Brain metastasis, Cancer immunotherapy, Cancer therapy, Chemotherapy resistance. “Others” group includes: Morphine relapse, Hepatic fibrosis, Autoimmune hepatitis, Sarcopenia, Rheumatoid arthritis, Diabetic wound healing, and Corneal Epithelial Healing. For *in vivo* analysis of EV dosage expressed as the number of EV per animal, publications were grouped into Cancer (as described above) and Others (including all other categories described above).

### Units’ conversion for EV loading and sncRNA-loaded EV doses *in vitro* and *in vivo*

To determine the amount of nucleic acids loaded into EVs across different exogenous methods, we first categorized studies based on their loading scheme: those reporting EV loading relative to total protein amount (µg) and those based on EV number. For each category, we calculated nucleic acid loading in picomoles (pmoles) per microgram of EVs or EV number, respectively.

siRNA and miRNA amounts (µg) were converted to pmoles using the following formulas: siRNA and miRNA amounts (µg) were converted to pmoles using the following formulas:

**Figure:**
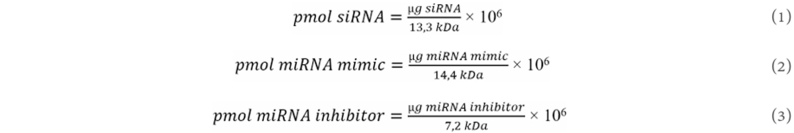


assuming 13.3, 14.4, and 7.2 kDa as average molecular weights for siRNA, miRNA mimics, and miRNA inhibitors, respectively.

For double-stranded RNA, optical density (OD) values were converted to pmoles based on the assumption that 1 OD = 50 µg/mL. Then, pmoles of siRNA and miRNA mimics were calculated using the formulas above.

In 14 studies where nucleic acid or EV concentrations were reported in µg/mL without a specified volume, a reaction volume of 100 µL was assumed.

Absolute quantification of nucleic acids loaded into EVs was expressed as nucleic acid (NA) copies per microgram of EV protein or per EV number. Copy numbers were calculated using Avogadro’s number, applying the formula: n. copies = mol × (6.022 × 10^23^).

*In vitro* EV dosage was expressed as micrograms of protein or number of nucleic acid (NA)-loaded EVs per cell. In 15 studies where the dosage was expressed as micrograms or number/mL, a volume of 2 mL was considered for a 6-well plate, 1 mL for a 12-well plate, 500 µL for a 24-well plate, and 100 µL for a 96-well plate. When treatment dosage was not reported or provided in micrograms, number, or volume of NA-loaded EVs without specifying cell number or surface area, the study was included in the “not reported” subgroup.

*In vivo* EV dosage was divided according to the unit of measurement in micrograms of protein or a number of NA-loaded EVs per animal. When EV dosage was expressed as mg/g without specifying the animal weight, an average weight of 19g was assumed for 5-week-old male BALB/c nude mice and 25g for 6- to 8-week-old NOD/SCID male mice in two studies, respectively.

When treatment dosage was not reported or provided as volume or mg/mL without specifying volume, the study was included in the “not reported” subgroup. For dosage analysis, only studies on mice were included.

## RESULTS

**Figure 2:**
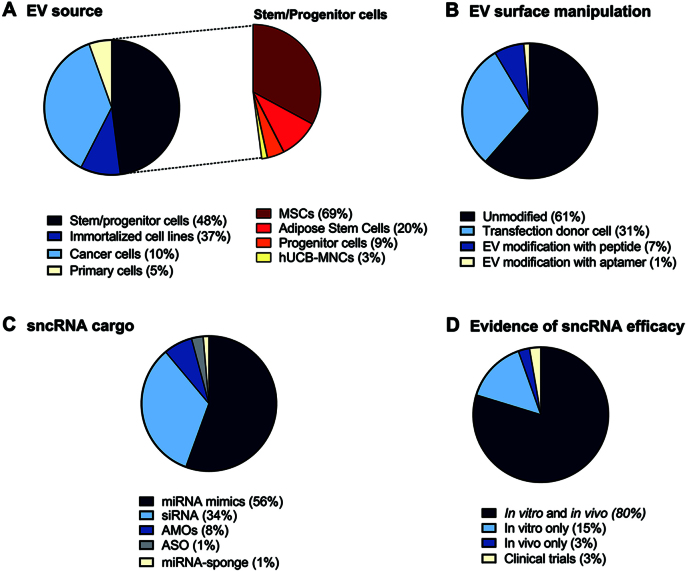
Overview of the main emerging features of EVs as sncRNA delivery systems in the 74 works analyzed. Pie charts illustrating the percentage (%) of EV donor cells used (A - the inset on the right represents a focus on the % of different stem cell types), surface engineering (B), type of non-coding RNA loaded (C), and evidence of sncRNA efficacy provided in the study (D). sncRNAs: Small non-coding RNAs; EVs: extracellular vesicles; EV: extracellular vesicle.

**Figure 3:**
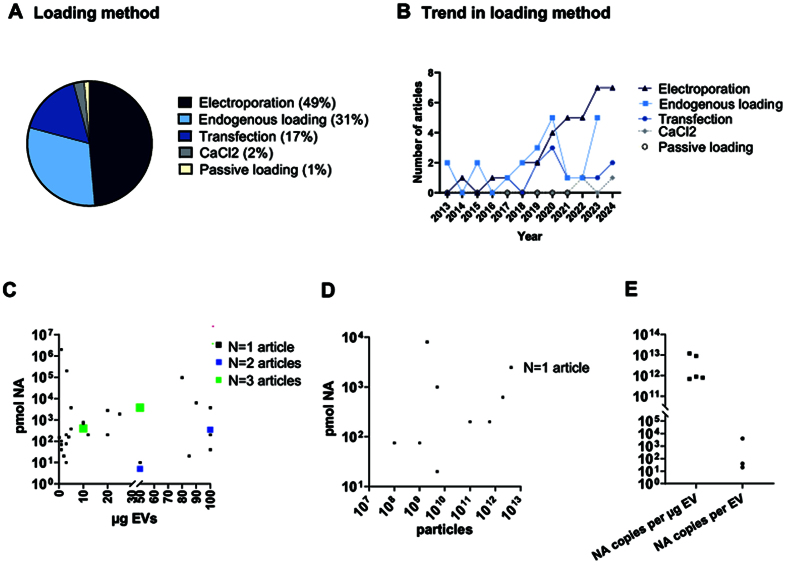
EV loading with snc RNA. (A) Pie chart illustrating the percentage of each loading method used across studies; (B) Graphical representation of the loading methods trend across years; (C and D) Graphs reporting the loading scheme based on pmol of loaded nucleic acid (NA) versus EVs µg (C) or EV number (D), for each analyzed article; (E) Graphical representation of the absolute quantification of cargo loading based on the number of copies of loaded NA per µg of EV or EV number (E). EV: Extracellular vesicle; sncRNAs: small non-coding RNAs; EVs: extracellular vesicles.

**Figure 4:**
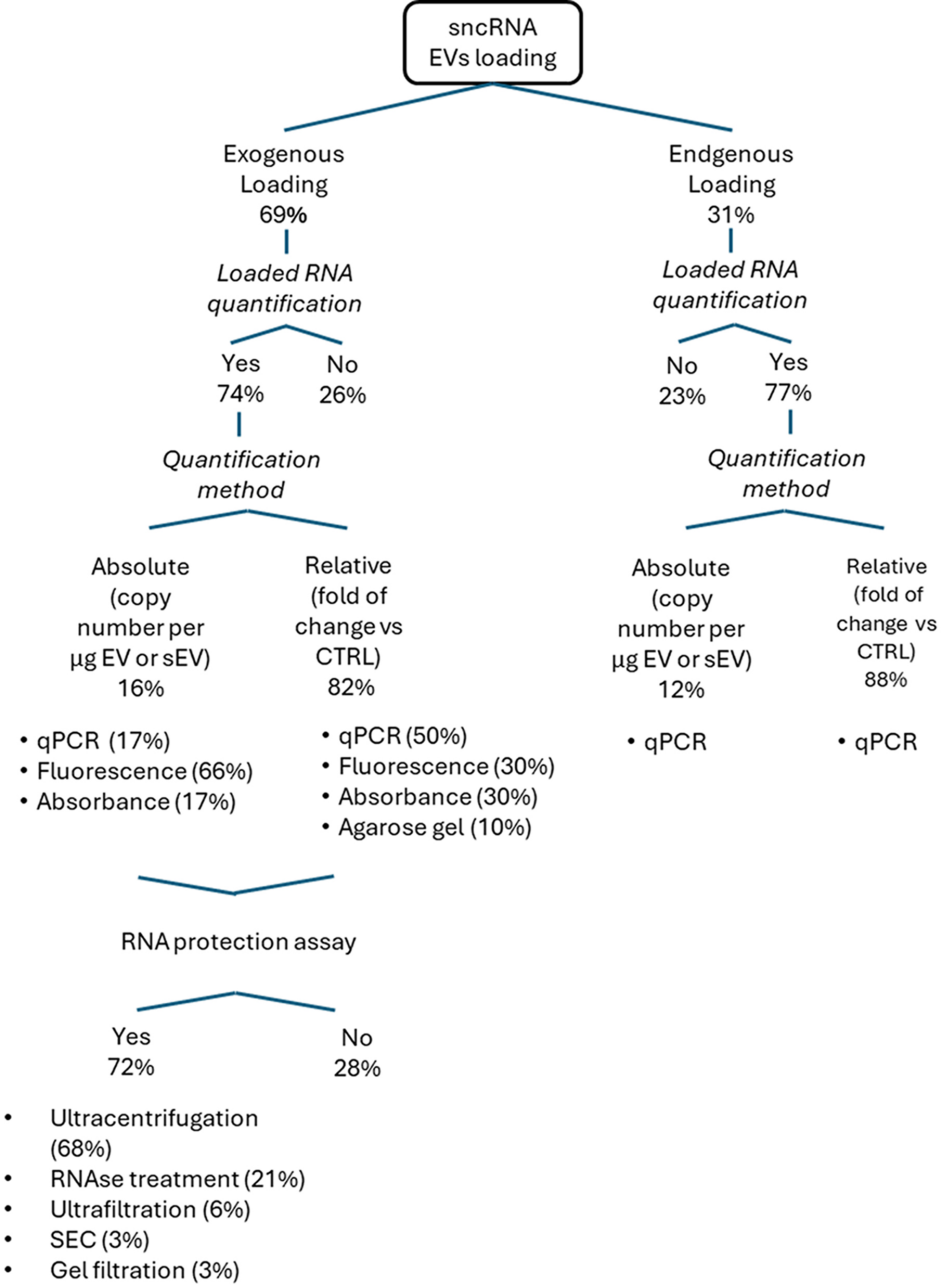
Flowchart illustrating EV loading and related nucleic acid quantification approaches. sncRNAs: Small non-coding RNAs; EVs: extracellular vesicles; EV: extracellular vesicle; qPCR: quantitative polymerase chain reaction; RNAse: ribonuclease; SEC: size exclusion chromatography.

**Figure 5:**
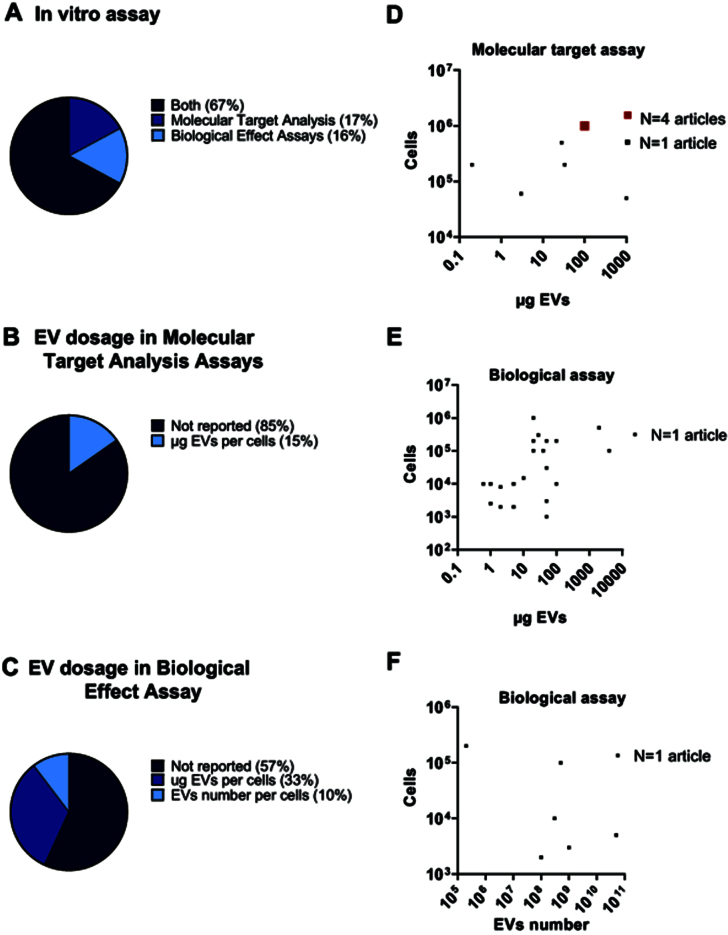
Analysis of sncRNA-loaded EV dosages *in vitro*. (A) Pie chart illustrating the percentage of studies that performed molecular target analysis assays, biological effect assays, or both *in vitro*; (B) Pie chart showing the distribution of EV dosages and measurement units used in molecular target analysis assays; (C) Pie chart illustrating the distribution of EV dosages and measurement units used in biological effect assays; (D) Graphical representation of doses based on protein quantification used in molecular target analysis assays; (E) Graphical representation of reported doses based on protein quantification used in biological effect assays; (F) Graphical representation of reported doses based on particle quantification used in biological effect assays. sncRNA: Small non-coding RNAs; EVs: extracellular vesicles; EV: extracellular vesicle.

**Figure 6:**
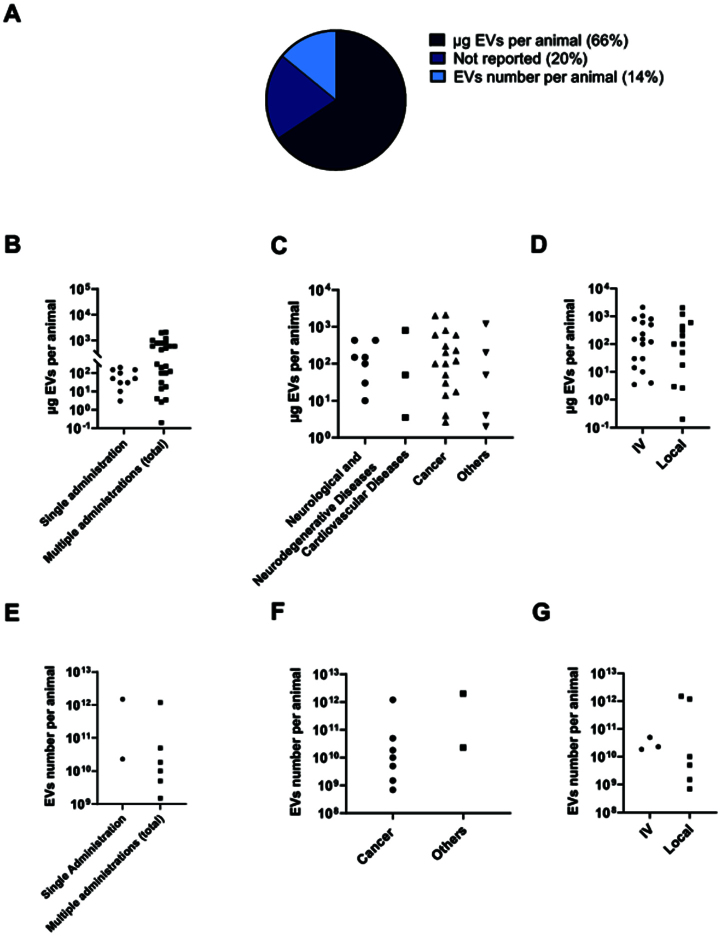
Analysis of sncRNA-loaded EVs dosages in preclinical studies. (A) Pie chart illustrating the distribution of EV dosages based on protein quantification, number of particles quantification, or not reported. (B-D) Graphical representations of reported EV doses based on protein quantification for single vs multiple administration (B), targeted disease (C), and route of administration (D). (E-G) Graphical representations of reported EV doses based on particle quantification for single vs multiple administration (E), targeted disease (F), and route of administration (G). sncRNA: Small non-coding RNAs; EVs: extracellular vesicles; EV: extracellular vesicle; IV: intravenous injection.

We selected and analyzed 74 studies on sncRNA-loaded EVs. The cell type used as an EV source, the EV isolation method employed, the type of sncRNA cargo, the experimental context used to assess sncRNA efficacy (e.g.*, in vitro*, *in vivo*, clinical trial), and the targeted disease are reported in Table 1. In detail, a comprehensive analysis was conducted on all aspects of EV development as a delivery system, encompassing production, engineering, and experimental context [Figure 2], loading strategies [Figures 3 and 4], *in vitro* dosages [Figure 5] and *in vivo* [Figure 6] dosages used to assess the efficacy and safety of the RNA-loaded nanoplatforms.

**Table 1:** Preclinical and clinical studies on sncRNA-loaded EVs as drug delivery systems analyzed in this review

**EV source**	**EV isolation method**	**sncRNA cargo**	**Evidence of sncRNA-loaded EV efficacy**	**Disease**	**Ref.**
Murine DCs	-Centrifugation:12,000 × *g* for 30 min; Ultracentrifugation: 120,000 × *g* for 1 h	siBACE1	*In vitro* and *in vivo*	Alzheimer’s disease	[22]
PANC-1	-Sucrose cushion ultracentrifugation at 100,000 × *g* for 90 min; -Centrifugation: 100,000 × *g* for 90 min	siPAK4	*In vitro* and *in vivo*	Pancreatic cancer	[23]
Expi293F	-Differential centrifugation: 500 × *g* for 10 min, 3,000 × *g* for 20 min, 10,000 × *g* for 30 min; -Filtration: 0.22 µm filter; -Ultracentrifugation: twice 130,000 × *g* for 70 min	siMMP13	*In vitro* and *in vivo*	Osteoarthritis	[24]
RAW264.7	-Differential centrifugation: 2,000 × *g* for 20 min, 10,000 × *g* for 30 min; -Filtration: 0.22 µm filter; -Ultracentrifugation: 110,000 × *g* for 70 min	siRIPK3	*In vitro* and *in vivo*	Autoimmune hepatitis	[25]
ADSCs	-Differential centrifugation: 300 × *g* for 10 min, 2,000 × *g* for 20 min, 10,000 × *g* for 30 min; -Filtration: 0.22 µm filter; -Ultracentrifugation: twice 100,000 × *g* for 1 h	siSAV	*In vitro* and *in vivo*	Myocardial infarction	[26]
Murine DCs	Serial centrifugation	siα-Syn	*In vitro* and *in vivo*	Parkinson’s disease	[27]
iNSC	-Differential centrifugation: 300 × *g* for 10 min, 3,000 × *g* for 20 min, 10,000 × *g* for 30 min; -Filtration: 0.22 µm filter; -Ultracentrifugation: twice 140,000 × *g* for 70 min	siCCL2	*In vitro* and *in vivo*	Spinal cord injury	[28]
HEK293T	-Differential centrifugation: 300 × *g* for 10 min, 3,000 × *g* for 20 min,10,000 × *g* for 30 min; -Ultracentrifugation: 120,000 × *g* for 70 min	siSOX2	*In vitro*	Lung cancer	[29]
HEK293T	Ultracentrifugation: 110,000 × *g* for 70 min	siHGF	*In vitro* and *in vivo*	Gastric cancer	[30]
BJ	-Differential centrifugation: 800 × *g* for 5 min, 2,000 × *g* for 10 min; -Filtration: 0.22 µm filter; -Ultracentrifugation: twice 100,000 × *g* for 2 h	siKrasG12D	*In vitro* and *in vivo*	Pancreatic cancer	[31]
HEK293 and MSCs	-Differential centrifugation: 300 × *g* for 10 min, 2,000 × *g* for 10 min; -Ultracentrifugation: twice 100,000 × *g* for 70 min	siPLK-1	*In vitro*	Bladder cancer	[32]
HEK293T	Exosome isolation kit (Invitrogen)	siMOR	*In vitro* and *in vivo*	Morphine relapse	[33]
HEK293T	-Differential centrifugation: 500 × *g* for 5 min, 10,000 × *g* for 30 min; -Ultracentrifugation: twice 100,000 × *g* for 60 min	siSIRT6	*In vitro* and *in vivo*	Prostate cancer	[34]
RAW 264.7	-Differential centrifugation: 300 × *g* for 10 min, 2,000 × *g* for 10 min, 10,000 × *g* for 30 min; -Filtration: 0.22 µm filter; -Ultracentrifugation: 100,000 × *g* for 70 min	siFGL1 and siTGF-β1	*In vitro* and *in vivo*	Cancer immunotherapy	[35]
HEK293T	Sequential centrifugation	siTPD52	*In vitro* and *in vivo*	Breast cancer	[36]
ADMSCs	Ultracentrifugation: 500,000 × *g* for 2 h	siNF-κB	*In vitro* and *in vivo*	Skin lesion	[37]
HEK293T	Polyethylene glycol (PEG) precipitation and ultracentrifugation	siDRAS-AS1	*In vitro* and *in vivo*	Triple-negative breast cancer	[38]
HEK293T	-Differential centrifugation: 500 × *g* for 10 min, 2,000 × *g* for 10 min, 10,000 × *g* for 30 min; -Filtration: 0.22 µm filter; -Ultracentrifugation: 140,000 × *g* for 2 h	siLPCAT1	*In vitro* and *in vivo*	Lung cancer brain metastasis	[39]
OCI-MY5	-Differential centrifugation: 300 × *g* for 10 min, 2,000 × *g* for 10 min, 10,000 × *g* for 30 min; -Ultracentrifugation: 100,000 × *g* for 90 min	siAIMP1	*In vitro* and *in vivo*	Multiple myeloma	[40]
MSCs	-Differential centrifugation: 2,000 × *g* for 20 min, 10,000 × *g* for 30 min; -Filtration: 0.22 µm filter; -Ultracentrifugation: 100,000 × *g* for 70 min	siSurvivin	*In vitro* and *in vivo*	Cancer therapy	[41]
HEK293T	Exosome Isolation Reagent (Invitrogen)	siKRAS	*In vitro* and *in vivo*	Lung cancer	[42]
HEK293T	-Differential centrifugation: 3,000 × *g* for 20 min, 10,000 × *g* for 20 min; -Ultracentrifugation: 110,000 × *g* for 80 min	sic-Met	*In vitro* and *in vivo*	Gastric cancer	[43]
bEND.3	Exosome Isolation Reagent (Invitrogen)	siVEGF	*In vitro* and *in vivo*	Brain cancer	[44]
SH-SY5Y	-Centrifugation: 400 × *g* for 5 min -Filtration: 0.22 µm filter; -Centrifugation: 21,460 × *g* overnight	siHSP27	*In vitro*	Neuroblastoma	[45]
MSCs	-Differential centrifugation: 800 × *g* for 5 min, 2,000 × *g* for 10 min; -Filtration: 0.22 µm filter; -Ultracentrifugation: twice 100,000 × *g* for 2 h	siKrasG12D	Phase I	Metastatic pancreas cancer with KrasG12D mutation	NCT03608631
MSCs	-Differential centrifugation: 800 × *g* for 5 min, 2,000 × *g* for 10 min; -Filtration: 0.22 µm filter; -Ultracentrifugation: 100,000 × *g* for 3 h	siSTAT3 or STAT3 ASO	*In vivo*	Hepatic fibrosis	[46]
hbmMSCs	-Differential centrifugation: 300 × *g* for 10 min, 2,000 × *g* for 15 min, 10,000 × *g* for 30 min; -Ultracentrifugation: twice 110,000 × *g* for 70 min	α-syn ASO	*In vitro* and *in vivo*	Parkinson’s disease	[47]
GL261	-Differential centrifugation: 12,000 × *g* for 20 min, 20,000 × *g* for 30 min; -Ultracentrifugation: 100,000 × *g* for 60 min	IGF-1R ASO	Phase I	Glioblastoma	NCT01550523
hUCB-MNCs	-Differential centrifugation: 2000 × *g* for 20 min, 10,000 × *g* for 30 min; -Ultracentrifugation: twice 100,000 × *g* for 2 h	miR-124-3p	*In vitro* and *in vivo*	Parkinson’s disease	[48]
HEK293	Exocib Exosomes Isolation kit	miR-365a-3p	*In vitro*	Hepatocellular carcinoma	[49]
MSCs	-Differential centrifugation: 2,000 × *g* for 20 min, 10,000 × *g* for 30 min; -Ultracentrifugation: 120,000 × *g* for 70 min	miR-26a	*In vitro* and *in vivo*	Hepatocellular carcinoma	[50]
MSCs	-Differential centrifugation: 300 × *g* for 10 min, 2,000 × *g* for 10 min, 10,000 × *g* for 30 min; -Ultracentrifugation: twice 120,000 × *g* for 70 min	miR-588	*In vitro* and *in vivo*	Triple-negative breast cancer	[51]
HEK293T	-Ultracentrifugation: 100,000 × *g* for 3 h, 100,000 × *g* for 1 h Or -Exoquick^-TC^kit (System Biosciences)	miR-21a-5p	*In vitro* and *in vivo*	Doxorubicin-induced cardiotoxicity	[52]
MG63	-Differential centrifugation: 800 × *g* for 5 min, 2,000 × *g* for 20 min; -Filtration: 0.22 µm filter; -Ultracentrifugation: twice 100,000 × *g* for 2 h	miR-665	*In vitro* and *in vivo*	Osteosarcoma	[53]
MDA-MB-231	PureExo® Exosome Isolation kit	miR-125	*In vitro* and *in vivo*	Lung cancer	[54]
HEK293T	exoEasy™ Exosome Isolation kit (Qiagen)	miR-26a	*In vitro*	Cancer therapy	[55]
HEK293T	-Differential centrifugation: 300 × *g* for 10 min, 1,000 × *g* for 30 min, 10,000 × *g* for 30 min; -Ultracentrifugation: twice 140,000 × *g* for 90 min	miR-34a	*In vitro*	Oral cancer	[56]
HEK293T	-Exoquick^-TC^ kit (System Biosciences)	miR-let7c-5p	*In vitro*	Breast cancer	[57]
ADMSCs	-Differential centrifugation: 300 × *g* for 5 min, 3,000 × *g* for 20 min, 6,000 × *g* for 40 min, 10,000 × *g* for 60 min; -Ultracentrifugation: twice 100,000 × *g* for 60 min	miR-101	*In vitro* and *in vivo*	Osteosarcoma	[58]
ADMSCs	-Differential centrifugation: 300 × *g* for 10 min, 2,000 × *g* for 10 min; -Filtration: 0.22 µm filter; -Ultrafiltration: 4,000 × *g* for 30 min using Amicon Ultra-15 centrifugal filter; -Sucrose/D_2_O cushion centrifugation: 100,000 × *g* for 60 min	miR-511-3p	*In vitro* and *in vivo*	Spinal cord injury	[59]
BMSCs	Serial centrifugation	miR-338-5p	*In vitro* and *in vivo*	Spinal cord injury	[60]
HEK-293 and r-BMSC	-Differential centrifugation: 300 × *g* for 10 min, 2,000 × *g* for 30 min, 10,000 × *g* for 60 min, 20,000 × *g* for 2 h; -Ultracentrifugation: 100,000 × *g* for 70 min	miR-29b	*In vitro* and *in vivo*	Alzheimer’s disease	[61]
DC2.4	-Differential centrifugation: 1,000 × *g* for 10 min, 10,000 × *g* for 30 min; -Filtration: 0.22 µm filter; -Ultracentrifugation: 100,000 × *g* for 70 min	miR-124	*In vitro* and *in vivo*	Cocaine-induced neuroinflammation	[62]
hucMSCs	Total exosome isolation reagent kit (Invitrogen)	miR-145-5p	*In vitro* and *in vivo*	Pancreatic ductal adenocarcinoma	[63]
HEK293	-Differential centrifugation: 2,000 × *g* for 15 min, 10,000 × *g* for 30 min; -Ultracentrifugation: twice 120,000 × *g* for 70 min	miR-1-3p	*In vitro* and *in vivo*	Esophageal cancer	[64]
BMSCs	PureExo® Exosome Isolation kit	miR-30c, miR-181b or miR-613	*In vitro* and *in vivo*	Lung cancer	[65]
BMSCs	Total exosome isolation reagent kit (Invitrogen)	miR-302	*In vitro* and *in vivo*	Myocardial infarction	[66]
hASC	-Differential centrifugation: 400 × *g* for 10 min, 2,000 × *g* for 15 min; -Filtration: 0.22 µm filter; -Ultrafiltration: 4,000 × *g* for 30 min using Amicon Ultra-15 centrifugal filter; -Ultracentrifugation: 100,000 × *g* for 70 min	miR-21-5p	*In vitro* and *in vivo*	Diabetic wound	[67]
iPSC	-Differential centrifugation: 300 × *g* for 10 min, 2,000 × *g* for 20 min, 10,000 × *g* for 30 min; -Filtration: 0.22 µm filter; -Ultracentrifugation: 100,000 × *g* for 70-120 min	miR-23b, miR-21-5p, miR-199b-5p	*In vitro* and *in vivo*	Spinal cord injury	[68]
MSC, HepG2 and SW1116	-Differential centrifugation: 300 × *g* for 10 min, 2,000 × *g* for 10 min, 10,000 × *g* for 30 min; -Ultracentrifugation: 100,000 × *g* for 70 min	miR-1270	*In vitro* and *in vivo*	Colorectal cancer	[69]
hUCMSCs	-Differential centrifugation: 300 × *g* for 10 min, 2,000 × *g* for 10 min, 10,000 × *g* for 30 min; -Ultracentrifugation: 100,000 × *g* for 70 min	miR-451a	*In vitro* and *in vivo*	Rheumatoid arthritis	[70]
hUCMSCs	-Differential centrifugation: 300 × *g* for 10 min, 2,000 × *g* for 10 min, 10,000 × *g* for 30 min; -Filtration: 0.22 µm filter; -Ultracentrifugation: 110,000 × *g* for 70 min	miR-34c-5p	*In vitro* and *in vivo*	Acute myeloid leukemia	[71]
L02, SMMC-772 and MHCC-97H	-Differential centrifugation: 500 × *g* for 5 min, 2,000 × *g* for 10 min; -Filtration: 0.22 µm filter; -Ultracentrifugation: twice 100,000 × *g* for 70 min	miR-26a	*In vitro* and *in vivo*	Hepatocellular carcinoma	[72]
ADSCs	-Differential centrifugation: 300 × *g* for 10 min, 3,000 × *g* for 10 min, 10,000 × *g* for 30 min; -Ultracentrifugation: twice 100,000 × *g* for 70 min	miR-24-3p	*In vivo*	Corneal epithelial healing	[73]
A549	-Differential centrifugation: 2,000 × *g* for 15 min, 16,000 × *g* for 20 min; -Ultracentrifugation: 100,000 × *g* for 90 min; -Filtration: 0.22 µm filter	miR-563	*In vitro* and *in vivo*	Lung cancer	[74]
HEK293T	-Differential centrifugation: 2,000 × *g* for 30 min, 10,000 × *g* for 30 min; -Ultracentrifugation: 100,000 × *g* for 4 h	miR-484	*In vitro* and *in vivo*	Chemotherapy resistance	[75]
ADSCs	-Differential centrifugation: 300 × *g* for 15 min, 3,000 × *g* for 15 min, 10,000 × *g* for 25 min; -Filtration: 0.22 µm filter; -Ultracentrifugation: 110,000 × *g* for 70 min	miR-654-5p	*In vitro* and *in vivo*	Hepatocellular carcinoma	[76]
MEFs	Total Exosome Isolation Reagent (Invitrogen)	miR-146a	*In vitro* and *in vivo*	Breast cancer	[77]
A375, SKMel-5, SKMel-28	-Differential centrifugation: 300 × *g* for 10 min, 2,000 × *g* for 10 min, 10,000 × *g* for 30 min; -Ultracentrifugation: 100,000 × *g* for 2 h	miR-195-5p	*In vitro* and *in vivo*	Melanoma	[78]
293T/17	-Differential centrifugation: 2,000 × *g* for 20 min, 10,000 × *g* for 30 min; -Filtration: 0.22 µm filter; -Ultracentrifugation: 10,000 × *g* for 70 min	miR-126	*In vitro* and *in vivo*	Periodontitis	[79]
ADSCs	-Differential centrifugation: 1,500 × *g* for 10 min, 2,000 × *g* for 10 min, 10,000 × *g* for 30 min; -Filtration: 0.22 µm filter; -Ultracentrifugation: 100,000 × *g* for 70 min; -Filtration: 0.22 µm filter; -Ultracentrifugation: 100,000 × *g* for 70 min	miR-132	*In vitro* and *in vivo*	Diabetic wound healing	[80]
HEK293	-Differential centrifugation: 2,000 × *g* for 20 min, 10,000 × *g* for 30 min; -Ultracentrifugation: 120,000 × *g* for 70 min	miR-let-7a	*In vitro* and *in vivo*	Breast cancer	[81]
ASCs	ExoQuick reagent (System Biosciences)	miR-125b	*In vitro* and *in vivo*	Hepatocellular carcinoma	[82]
SMSCs	-Differential centrifugation: 300 × *g* for 15 min, 2,000 × *g* for 15 min; -Filtration: 0.22 µm filter; -Ultrafiltration: 4,000 × *g* for 30 min using Amicon Ultra-15 centrifugal filter; -Sucrose/D_2_O cushion centrifugation: 100,000 × *g* for 60 min	miR-126-3p	*In vitro* and *in vivo*	Wound healing	[83]
AMSCs	ExoQuick-TC Kit (System Biosciences)	miR-122	*In vitro* and *in vivo*	Hepatocellular carcinoma	[84]
MSCs	ExoQuick-TC Kit (System Biosciences)	miR-146b	*In vitro* and *in vivo*	Glioma	[85]
EPCs	-Differential centrifugation: 300 × *g* for 15 min, 2,000 × *g* for 30 min, 20,000 × *g* for 70 min; -Ultracentrifugation: 170,000 × *g* for 90 min	miR-210	*In vitro*	Hypoxia/Reoxygeneation-injury	[86]
BMSCs	ExoQuick-TC Kit (Invitrogen)	miR-125b	*In vitro* and *in vivo*	Myocardial ischemia reperfusion injury	[87]
MSCs	ExoQuick-TC Kit (System Biosciences)	AMO-142-3p	*In vitro* and *in vivo*	Breast cancer	[88]
cbMSCs	-Differential centrifugation: 300 × *g* for 10 min, 2,000 × *g* for 10 min, 10,000 × *g* for 30 min; -Ultracentrifugation: twice 100,000 × *g* for 70 min	AMO-221	*In vitro* and *in vivo*	Colonrectal carcinoma	[89]
HEK293T	exoEasy Maxi kit (Qiagen)	AMO-21	*In vitro* and *in vivo*	Glioblastoma	[90]
HUVECs	-Differential centrifugation: 1,500 × *g* for 15 min, 10,000 × *g* for 30 min; -Serial filtration: 0.45 µm and 0.22 µm filter; -Serial ultracentrifugation: 100,000 × *g* for 70 min, 100,000 × *g* for 60 min, 100,000 × *g* for 70 min	antagomiR-BART10-5p and antagomiR-18a	*In vitro* and *in vivo*	Cancer angiogenesis	[91]
BMSCs	-Differential centrifugation: 3,000 × *g* for 30 min, 30,000 × *g* for 60 min; -Ultracentrifugation: twice 120,000 × *g* for 2 h	antagomiR-467a-3p or antagomiR-874-5p	*In vitro* and *in vivo*	Sarcopenia	[92]
HEK293T	-Differential centrifugation: 300 × *g* for 10 min, 2,000 × *g* for 10 min, 10,000 × *g* for 30 min, 20,000 × *g* for 60 min; -Ultracentrifugation: 100,000 × *g* for 70 min	miR-21-sponge	*In vitro* and *in vivo*	Glioblastoma	[93]

sncRNAs: Small non-coding RNAs; EVs: extracellular vesicles; EV: extracellular vesicle.

Based on the origin of EVs, we grouped the EV donor cell types into four different categories [Figure 2A, left]. Among all the analyzed studies, EVs derived from stem and progenitor cells (48%) and immortalized cell lines (37%) were the most commonly used cell types. Given the predominant use of stem/progenitor cells, we further classified this category based on the specific cell type [Figure 2A, right]. Among these, mesenchymal stem cells (MSCs) were the overwhelmingly preferred source, representing 69% of the stem/progenitor-derived EV studies. Adipose-derived stem cells followed at 20%, while progenitor cells and human umbilical cord blood mononuclear cells (hUCB-MNCs) accounted for 9% and 3%, respectively. Cancer cell-derived EVs accounted for 10% and primary cell-derived EVs were the least represented (5%).

Regarding EV surface modification strategies [Figure 2B], the majority of studies (61%) employed unmodified, naïve EVs. Genetic engineering of donor cells, typically involving transfection to express fusion proteins or targeting ligands, was used in 31% of the studies. Post-isolation modifications were less common: 7% of studies used peptide-based surface engineering, and only 1% employed aptamer conjugation.

We further analyzed the types of sncRNA molecules loaded into EVs across studies. As reported in Figure 2C, miRNA mimics constituted the most frequently loaded sncRNA cargo, used in 56% of the studies. siRNAs were the second most common cargo type (33%), reflecting the popularity of RNA interference strategies. In contrast, antisense technologies were underrepresented: AMOs were used in 7% of studies, ASOs in 3%, and miRNA sponges in only 1%.

Finally, we evaluated the therapeutic outcomes [Figure 2D]. A minority of studies (15%) focused exclusively on *in vitro* experiments or directly *in vivo* (3%), after assessing *in vitro* the uptake and biocompatibility of naïve EVs. The majority of studies (80%) included both *in vitro* and *in vivo* experiments allowing for a basic, initial molecular characterization in *in vitro* models, followed by validation in *in vivo* systems that better mimic physiological conditions. Despite substantial evidence supporting the therapeutic potential of EV-based RNA therapeutics *in vivo*, only two ongoing clinical trials (3%) have been identified that investigate the safety and/or efficacy of EV-based sncRNA therapies.

### The issue of sncRNA loading into EVs

Effective and efficient loading of therapeutic agents within EVs is a crucial aspect in the development of EV-based therapeutics. Approaches are classified into two main categories: endogenous loading and exogenous loading. Endogenous loading, or pre-isolation loading, involves transfection of the donor cells to express the desired nucleic acid, protein, or peptide, which is incorporated into EVs during their biogenesis. In contrast, exogenous loading refers to post-isolation methods where cargo is introduced into pre-isolated EVs by passive loading (simple incubation of EVs with cargo), transfection, or using approaches that enhance membrane permeability, such as electroporation, sonication, and freeze-thaw cycles^[94]^.

We analyzed the different EV loading approaches used across studies [Figure 3A] and their trends over time [Figure 3B] to assess whether the most commonly used methods also exhibited growth trends. Electroporation and endogenous loading emerged as the two most widely used strategies for EV loading. Electroporation is the most widely used method (49%), and displays an evident growing trend in the last 5 years. It employs an electrical field to create transient pores in the EV membrane, allowing cargo entry. Despite its efficiency, electroporation poses challenges such as cargo aggregation, variability in loading efficiency, and sensitivity to protocol conditions^[95]^. The second most common method (31%) is endogenous loading, which relies on genetic modification of donor cells to naturally incorporate the desired nucleic acid into EVs during biogenesis. This strategy ensures high biocompatibility and stability of the cargo. However, it is inherently limited by cellular mechanisms, which limit the exact quantity of nucleic acid loaded, potentially resulting in lower and more variable loading efficiencies compared to exogenous methods^[96]^. Other methods, including EV transfection, CaCl_2_-mediated transfection, and passive loading, exhibit lower use rates and no increase in use over time. Direct EV transfection (17%), using lipid-based or chemical reagents (such as Lipofectamine, Exo-Fect, or ExoFectin), offers a simple and equipment-free alternative. However, it is limited by reagent availability and cost, and potential cytotoxicity. Alternatively, CaCl_2_ transfection has been developed to improve loading efficiency, especially for miRNAs^[97]^. It has been found to be a convenient and highly efficient method, although it is currently rarely used (2%). Passive loading, despite being the simplest method, remains the least adopted (1%). The major drawback is the lower loading efficiency compared to other methods, since no additional forces beyond sncRNA diffusion are used to facilitate EV membrane passage.

We next evaluated in detail the loading protocols, particularly focusing on the ratio of nucleic acid (NA) expressed in pmol per µg of EVs [Figure 3C] or per EV number [Figure 3D] used in each protocol. The majority of studies rely on EV quantification based on protein content [Figure 3C], although it is an indirect and not recommended quantification method^[13]^. The data distribution [Figure 3C] shows a remarkable variability across different studies. The papers tend to cluster in two opposite strategies: 10-1,000 pmol NA loaded into 1-10 µg EVs, or 10-100 pmol NA loaded into 50-100 µg EVs. A minority exceeded 10^3^ pmol loaded into a wide range (10 to 100 µg EVs). Despite the overall variability, we found four loading schemes adopted multiple times across studies (Figure 3C, points in green and blue), suggesting that specific loading protocols are consistently used and may serve as benchmarks for future standardization. An even greater variability is evident for loading approaches based on pmol NA per number of EVs [Figure 2D], with 10-10,000 pmol NA used for EV numbers ranging from 10^7^ to 10^13^ particles, indicating a lack of a loading protocol with a well-defined quantity of moieties to be used. The data distribution, along with the limited number of studies using this measurement unit, hinders the identification of clear and consistent trends across studies.

The absolute quantification of NA loaded into EVs was reported in only eight studies [Figure 3E], expressed either as NA copies per microgram of EV protein or per EV particle. A high degree of variability, spanning over one order of magnitude, was observed across studies in both cases. The reported values for NA copies per microgram of EV protein ranged from approximately 10^11^ to 10^14^ copies, while NA copies per EV particle spanned from 30-50 to 10^5^ copies.

To critically evaluate and rationalize these inconsistencies, we categorized loading approaches into exogenous (69%) and endogenous (31%) loading and detailed the methodologies employed for RNA quantification, as reported in Figure 4.

Loading efficiency in drug carrier systems is typically assessed using direct or indirect methods. Indirect methods estimate loading efficiency by measuring the amount of unincorporated sncRNA in the supernatant after centrifugation, often through fluorescence or absorbance measurements. For exogenous loading, RNA quantification was performed in 74% of studies, whereas 26% did not quantify the loaded RNA. Among studies that performed quantification, the majority (82%) reported relative measurements (fold change compared to unloaded control), while only 16% provided absolute values (copy number per µg of EVs or single EV). Notably, fluorescence-based methods accounted for the majority (66%) of absolute quantifications, followed by quantitative polymerase chain reaction (qPCR) (17%) and absorbance-based approaches (17%). For relative quantifications, qPCR was the most commonly used technique (50%), followed by fluorescence-based methods (30%), absorbance-based methods (30%), and agarose gel analysis (10%).

In the case of endogenous loading, 77% of studies quantified loaded RNA, with a higher proportion (88%) relying on relative quantification rather than absolute quantification (12%). Interestingly, qPCR was the exclusive method used for both absolute and relative quantification in these studies. Given the prevalence of indirect quantification methods and the high values observed in absolute quantification [Figure 3E and Figure 4], we further considered whether RNA protection assays were performed to ensure the removal of unloaded RNA after the loading process. Interestingly, 28% of the studies did not perform any RNA protection assays, neglecting potential contamination of unloaded RNA in EV preparation and errors in quantification. Among studies that performed RNA protection, most relied on ultracentrifugation (68%) to remove unbound RNA. Although ultracentrifugation is considered a gold standard for isolating and concentrating EVs, as demonstrated in Table 1, it fails to fully purify EVs from larger vesicles, proteins, and other contaminants^[98]^. As a result, ultracentrifugation may not completely separate loaded RNA from non-loaded RNA, and some unbound RNA may remain in the pellet, leading to an overestimation of the actual RNA content loaded into the EVs. Interestingly, RNase treatment was employed in only 21% of studies, despite its effectiveness in degrading unincorporated RNA and improving the accuracy of RNA loading assessment.

### sncRNA-loaded EVs dosages in in vitro preclinical assays

*In vitro* experiments are a crucial starting point for demonstrating the therapeutic potential of EVs. They enable an initial understanding of molecular mechanisms and provide a comprehensive basis for subsequent *in vivo* experiments. To evaluate and compare EV dosages used in *in vitro* treatments across studies, we first categorized the assays in two main groups: those assessing the efficacy based on molecular target analysis (i.e., PCR of nucleic acid targets and/or Western Blot of the related protein product) and those assessing the RNA therapeutic biological effect (e.g., cell viability, apoptosis, proliferation, migration assays, colony formation assay, tube formation assay, wound healing assay). We then analyzed the EV dosage used in each category.

As shown in Figure 5A, the majority of studies (67%) conduct both molecular target analysis and biological effect assays, while a smaller proportion focused exclusively on either molecular target analysis (17%) or biological effect assays (16%). This distribution suggests a trend toward correlating molecular changes with functional outcomes, providing a more comprehensive understanding of EV-mediated effects. Nevertheless, a significant issue is the lack of precise information on the EV quantities used in treatments [Figures 5B and 4C]. Indeed, 85% and 57% studies did not report the dosage used for molecular target analysis or biological effect assays, respectively.

For molecular target analysis, most studies used 100 µg EVs for 10^6^ cells [Figure 5D]. In contrast, the range of dosages reported for biological effect assays is more variable. Most studies reported dosages ranging from around 1 to 100 μg EVs per 10^3^-10^4^ cells, with some studies using up to 100 μg for 10^5^-10^6^ cells, and in a few cases, even reaching up to 1,000 μg [Figure 5E]. Only rarely did studies rely on EV number [Figure 5F], but the highly scattered distribution of reported dosages prevents the identification of a benchmark dose.

### sncRNA-loaded EV dosages in *in vivo* studies

Well-designed *in vivo* studies are crucial for advancing EV-based therapies toward clinical applications. These models allow for the evaluation of multiple aspects of EV applicability, including dosing, administration routes, therapeutic efficacy, and potential side effects, and contribute to a deeper understanding of EV-based therapy potential. For a better comparison of EV dosing strategies, we first categorized the analyzed studies based on the quantification method used (e.g., μg of EV protein or EV number per animal) and further analyzed dosing strategies with respect to administration frequency, targeted disease, and route of administration.

The majority of the analyzed studies quantified the EVs dose administered *in vivo* based on the total amount of protein (66%), whereas only a smaller proportion relied on EV number quantification (14%) and of note, the 20% of the studies did not report the exact dosage used [Figure 6A]. A first categorization of studies by single versus multiple administration revealed that the dose of EV protein from single-dose treatments was equal to cumulative dose from multiple administrations in a considerable (~50%) number of reports, while in the remaining cases, the cumulative doses were greater than the single ones [Figure 6B]. Within the multiple administration group, cumulative doses varied widely across studies, ranging from nanograms to over 10^3^ micrograms per animal, highlighting the lack of a standardized dosing approach. We further investigated whether the dosage regimen varied depending on the targeted disease. However, the data showed a higher variability within the same disease category than between different diseases. Regardless of disease, doses spanned from 10 to 1,000 μg EV protein per animal [Figure 6C] or 10^9^ to 10^12^ particles per animal [Figure 6F] , and no clear correlation was observed between dosage and the targeted tissue. Finally, we questioned whether the route of administration (systemic or local) influenced the dosage of EVs used [Figure 6D and G]. In the present studies, no higher dosage was used for systemic administration compared to local delivery. EV number-based dosing is used in a minority of studies. Overall, it appears that lower doses of EVs are typically administered in studies using multiple administrations and local administrations compared to single-dose and intravenous administration, respectively [Figure 6E and G]. However, the limited number of studies does not allow for robust conclusions regarding dosing strategies.

## DISCUSSION

EVs hold great potential as RNA drug delivery vehicles. Over the past decade, EV research has advanced considerably, particularly in understanding the biological properties of EVs and exploring their use in delivering sncRNAs. Preclinical studies have provided encouraging evidence of therapeutic efficacy, reinforcing the potential of EVs as promising bioinspired carriers for RNA-based therapy. However, despite these promising advances, clinical translation remains limited by technical, methodological, and regulatory challenges. Through a structured and in-depth analysis of the selected studies, this systematic review provides a comprehensive and critical overview of the EV-based RNA delivery landscape, highlighting key methodological trends and gaps, and translational challenges that currently hinder its widespread clinical application.

One of the key aspects in the development of EV-based therapeutics is the choice of the cellular source, as it influences EV yield, composition, and biodistribution^[99,100]^. Stem cell-derived EVs are widely employed due to their inherent regenerative and immunomodulatory properties^[101]^, offering a promising cell-free therapy in regenerative medicine. These EVs contain bioactive molecules that promote tissue repair, neuroprotection, and angiogenesis, making them highly attractive for therapeutic applications^[102]^. Among the various stem cell sources, mesenchymal stem cells (MSCs) emerged as the most frequently used for EV production in the selected studies. Their use spanned a wide range of disease models, both oncological and non-oncological, reflecting their acceptable safety and tolerability profiles, and promising therapeutic potential. To date, more than 80 studies have been registered investigating the clinical application of MSC-derived EVs^[103]^. The growing clinical interest in MSCs is further underscored by RYONCIL (remestemcel-L-rknd), the first FDA-approved MSC-based therapy for treating steroid-refractory acute graft-versus-host disease (SR-aGVHD) in pediatric patients aged 2 months and older. This milestone paves the way for the development and clinical translation of MSC-derived EVs. Moreover, adipose-derived stem cells (ADSCs) are increasingly used as a source of EVs for regenerative medicine applications, particularly in the context of skin repair. ADSC-derived EVs (ADSC-EVs) mimic the pro-regenerative functions of their parent cells, without the risks associated with stem cell transplantation, such as immune rejection or tumorigenicity^[104]^. For example, Lu *et al.* developed engineered ADSC-EVs encapsulating NF-κB siRNA for the treatment of inflammatory skin lesions, reporting enhanced wound closure and reduced local inflammation *in vivo*^[37]^. Similarly, Lv *et al.* showed that miR-21-5p-loaded ADSC-EVs significantly accelerated diabetic wound healing, promoting angiogenesis and re-epithelialization in a murine model^[67]^. Beyond skin applications, Sun *et al.* functionalized miR-24-3p-enriched ADSC-EVs into a hydrogel scaffold for corneal epithelial repair, demonstrating enhanced cell migration and tissue restoration *in vitro* and *in vivo*^[73]^. However, stem cell-derived EV manufacturing suffers from a lack of standardization in isolation protocols and characterization, leading to inconsistencies in EV purity and yield, and posing a challenge for large-scale production^[105]^. In contrast, immortalized cell lines provide a robust and scalable platform for EV production. They provide high yields, reproducibility, and easy genetic modification, making them ideal for engineering EVs with therapeutic cargos or surface modifications^[106]^. Nevertheless, their potential toxicity and immunogenicity may limit their clinical translation. Cancer cell-derived EVs were used predominantly in studies focusing on the delivery to the tumor itself, as they exhibit an intrinsic homotypic tropism, enabling tumor-targeted delivery, potentially increasing specificity and reducing off-target effects. Nonetheless, safety concerns regarding oncogenic cargo and the potential risk of tumorigenicity persist^[107]^. Primary cell-derived EVs were the least frequently used, possibly due to limited cell availability, difficulties in cell isolation, and low proliferation rates, which hinder large-scale EV production^[108]^. These percentages align with trends reported in the broader EV research field, where preclinical and clinical studies consistently identify stem cell-derived EVs as the most commonly utilized source, highlighting their favourable safety profile and therapeutic efficacy^[109-112]^.

One of the key aspects in the development of EV-based therapeutics is the choice of the cellular source, as it influences EV yield, composition, and biodistribution^[99,100]^. Stem cell-derived EVs are widely employed due to their inherent regenerative and immunomodulatory properties^[101]^, offering a promising cell-free therapy in regenerative medicine. These EVs contain bioactive molecules that promote tissue repair, neuroprotection, and angiogenesis, making them highly attractive for therapeutic applications^[102]^. Among the various stem cell sources, mesenchymal stem cells (MSCs) emerged as the most frequently used for EV production in the selected studies. Their use spanned a wide range of disease models, both oncological and non-oncological, reflecting their acceptable safety and tolerability profiles, and promising therapeutic potential. To date, more than 80 studies have been registered investigating the clinical application of MSC-derived EVs^[103]^. The growing clinical interest in MSCs is further underscored by RYONCIL (remestemcel-L-rknd), the first FDA-approved MSC-based therapy for treating steroid-refractory acute graft-versus-host disease (SR-aGVHD) in pediatric patients aged 2 months and older. This milestone paves the way for the development and clinical translation of MSC-derived EVs. Moreover, adipose-derived stem cells (ADSCs) are increasingly used as a source of EVs for regenerative medicine applications, particularly in the context of skin repair. ADSC-derived EVs (ADSC-EVs) mimic the pro-regenerative functions of their parent cells, without the risks associated with stem cell transplantation, such as immune rejection or tumorigenicity^[104]^. For example, Lu *et al.* developed engineered ADSC-EVs encapsulating NF-κB siRNA for the treatment of inflammatory skin lesions, reporting enhanced wound closure and reduced local inflammation *in vivo*^[37]^. Similarly, Lv *et al.* showed that miR-21-5p-loaded ADSC-EVs significantly accelerated diabetic wound healing, promoting angiogenesis and re-epithelialization in a murine model^[67]^. Beyond skin applications, Sun *et al.* functionalized miR-24-3p-enriched ADSC-EVs into a hydrogel scaffold for corneal epithelial repair, demonstrating enhanced cell migration and tissue restoration *in vitro* and *in vivo*^[73]^. However, stem cell-derived EV manufacturing suffers from a lack of standardization in isolation protocols and characterization, leading to inconsistencies in EV purity and yield, and posing a challenge for large-scale production^[105]^. In contrast, immortalized cell lines provide a robust and scalable platform for EV production. They provide high yields, reproducibility, and easy genetic modification, making them ideal for engineering EVs with therapeutic cargos or surface modifications^[106]^. Nevertheless, their potential toxicity and immunogenicity may limit their clinical translation. Cancer cell-derived EVs were used predominantly in studies focusing on the delivery to the tumor itself, as they exhibit an intrinsic homotypic tropism, enabling tumor-targeted delivery, potentially increasing specificity and reducing off-target effects. Nonetheless, safety concerns regarding oncogenic cargo and the potential risk of tumorigenicity persist^[107]^. Primary cell-derived EVs were the least frequently used, possibly due to limited cell availability, difficulties in cell isolation, and low proliferation rates, which hinder large-scale EV production^[108]^. These percentages align with trends reported in the broader EV research field, where preclinical and clinical studies consistently identify stem cell-derived EVs as the most commonly utilized source, highlighting their favourable safety profile and therapeutic efficacy^[109-112]^.

EVs have intrinsic tropism toward specific organs or diseased sites depending on their surface protein and lipid composition^[113]^. However, their innate targeting properties are limited, and several EV surface engineering strategies have been explored to enhance EV targeting ability and promote internalization into specific cells of interest^[114]^. Despite the potential advantages of these strategies in improving delivery precision, the majority of analyzed studies relied on naïve, unmodified EVs. Although surface modifications may compromise the function and structure of EVs or potentially trigger immune responses and reduce biocompatibility, we believe that the main deterrent is the technical difficulty required for EV surface functionalization. Surface engineering can be broadly categorized into two strategies: pre-isolation, through genetic manipulation of donor cells, or post-isolation, using physical or chemical modifications. Genetic engineering of donor cells, typically achieved by expressing fusion proteins that link EV membrane proteins to the targeting moiety, emerged as the most commonly employed method; indeed, this method ensures stable and uniform surface modification^[115]^. However, this approach requires cell line engineering, which may limit its applicability in clinical settings, and it is limited only to protein or peptide conjugation^[116]^. Post-isolation methods enable the conjugation of a broader range of molecules, including aptamers, and the simultaneous functionalization of EVs derived from different cell sources, making them more versatile strategies. Nevertheless, these approaches require additional purification steps and may negatively affect EV yield and integrity^[114]^.

The predominant RNA cargo is represented by miRNA mimics and siRNA, while miRNA inhibitors, including AMOs and miRNA sponges, and ASOs were used less frequently. The reasons why miRNA inhibitors are so less represented than miRNA mimics are not obvious, given the promising therapeutic potential of both these molecules^[117]^. On the other hand, for microRNA sponges, an issue could be that they are long, elaborately designed RNA transcripts transcribed from strong promoters by DNA-based vectors^[118]^. Therefore, their complex mechanism of action and design, along with their larger size, might limit their efficient packaging and delivery within EVs. Finally, a possible explanation for the limited number of studies utilizing EVs to encapsulate ASOs for delivery research could be that most ASOs, especially those nearing clinical application, have modified and stabilized backbones^[119]^, reducing the urgency for the development of protective carriers.

A critical barrier to the clinical translation of EV-based RNA therapeutics lies in the lack of standardized RNA loading protocols and quantification strategies. This consideration emerged after we made a substantial effort to harmonize the units of measurement for both RNA and EV quantities across the different loading protocols [Figure 3C-E]. Our analysis highlighted a substantial variability in the protocols adopted for RNA loading into EVs, particularly in the amount of nucleic acid used per unit of EVs. This inconsistency is related to a lack of standardized metrics for EV quantification, which remains one of the major technical challenges in the field. EV quantification is commonly based on either total protein content or particle number, but the correlation between these metrics is not always trivial^[21]^. Importantly, most studies report either one of these units, but not both, limiting the feasibility of a comprehensive cross-study comparison. Protein-based quantification, although widely used, is an indirect measure of EV content. It can be significantly influenced by several biological and technical factors, including the cell source, passage number, culture conditions, and the isolation method used, and does not always correlate with functional EV activity^[120]^. Different techniques, such as ultracentrifugation, precipitation, and density gradient methods, co-isolate contaminants such as protein aggregates and lipoproteins, impacting the accuracy of protein-based EV quantification, often leading to an overestimation. Alternative approaches for EV quantification include nanoparticle tracking (NTA), tunable resistive pulse sensing (TRPS), or nano-flow cytometry (nFCM)^[121,122]^. These technologies can count and size particles within certain ranges, using calibrators and reference materials. The quantification of particle number in EV preparations depends on the technique used for measurement, as each method has a specific range of sizes and concentrations that enable accurate quantification. The choice of calibrators and the lack of appropriate reference materials limit the full quantitative potential of these instruments, often requiring data extrapolation outside of the calibrated quantification range, leading to higher uncertainties^[123]^. In light of these considerations, an orthogonal analysis, combining different techniques, is recommended to address these challenges and improve the accuracy and reproducibility of EV quantification measurements^[122]^.

The variability in assessing loaded RNA is another issue. High variability in the absolute quantification of nucleic acids loaded per single EV can be ascribed to the different efficiency of the used loading methods (electroporation, transfection, or endogenous loading in the 8 studies). However, different efficiencies were observed even within the same loading method. For instance, Xu *et al.* and Zhang *et al.* reported a loading efficiency of 5-47 and 4,113 ± 102.6 siRNA molecules, respectively, using the electroporation method^[23,24]^. Electroporation is a commonly used technique that leads to higher EV loading capacity compared with other methods; however, it is characterized by high variability among the laboratories. Technical parameters such as voltage, pulse width, and number of pulses applied should be clearly documented, along with information about the buffer composition and the type of cuvettes or vials used. These may influence loading efficiency and vesicle integrity^[124]^. For instance, higher voltage and longer pulse durations can increase pore formation in the EV membrane, potentially increasing cargo loading. However, excessively augmenting these parameters can damage the EVs, reducing their integrity and functionality. Notably, an intermediate electroporation condition (750 V, 10 pulses) resulted in more efficient miRNA transfer compared to the highest tested setting (1,000 V, 10 pulses), suggesting that moderate parameters may be more effective for functional EV loading^[125]^. In addition, the material of the electrodes can also influence loading efficiency; for instance, aluminium electrodes may release aluminium ions (Al^3+^) during electroporation, which induce siRNA aggregation and reduce encapsulation efficiency^[126]^.

Another explanation for such dispersion of data is that the different methodologies used for NA quantification into EVs are substantially inconsistent with one another. Variability in loading efficiency, often assessed through relative rather than absolute quantification, limits accurate dosing calculation and reproducibility. Most studies rely on fluorescence and absorbance-based methods to quantify the RNA concentration loaded into EVs. One common approach involves the fluorescence measurement of a fluorolabelled NA before and after ultracentrifugation, estimating the loaded NA based on the difference in fluorescence intensity between the pellet, which is assumed to contain only the loaded vesicles, and the supernatant, which retains the unincorporated NA. In the most quantitative asset, this method can use a standard curve of fluorolabelled NA to interpolate unknown values, and the results can be further validated by qPCR analysis. This method provides a more direct quantification of amplifiable NA copies but still depends on the accuracy of the standard curve. Similarly, absorbance-based methods utilize comparable principles, measuring the difference in absorbance to derive RNA incorporation.

While these fluorescence and absorbance-based techniques are widely used, they provide indirect estimations of RNA loading. These methods assume that all detected signals correspond to successfully encapsulated RNA, without accounting for surface adsorption, incomplete loading, degradation, and inefficient separation of unloaded molecules. qPCR-based quantification provides a more specific and reliable measure of encapsulated RNA, helping to overcome some of the aforementioned limits, especially the degradation issues. However, it requires RNA extraction and reverse transcription processes that may lead to potential RNA loss. Additionally, qPCR relies on expensive primer-probe assays customized for the detection of the loaded RNA, especially applied to small RNAs such as siRNA or miRNA. Most studies utilize relative qPCR, which indirectly estimates the RNA loading efficiency by comparing RNA expression in loaded versus unloaded EVs, often normalized to a reference gene. In contrast, absolute qPCR provides a direct, absolute quantification of the targeted RNA, but requires the establishment of a standard curve to extrapolate unknown values. Digital PCR (dPCR) offers a promising alternative, enabling absolute quantification of RNA molecules with high sensitivity and reproducibility. Unlike qPCR, dPCR does not rely on standard curves, reducing variability and improving quantification accuracy, particularly for low-abundance targets^[127]^. However, dPCR requires specialized and expensive instrumentation, limiting its accessibility in many laboratories. An alternative approach could be the use of advanced imaging techniques such as Stochastic Optical Reconstruction Microscopy (STORM). By employing fluorophore-labelled RNA, STORM enables the precise quantification of loaded RNA through colocalization analysis, without interfering with the integrity of the EVs^[128,129]^. This approach not only preserves the integrity of the loaded vesicles but also provides a more accurate and direct quantification of RNA cargo. Nevertheless, this technique requires specialized expertise and costly equipment, as well as well-optimized data analysis workflows, to minimize artifacts and ensure reliable quantification. In addition, cryo-transmission electron microscopy (cryo-TEM) has gained attention as a powerful tool to visualize EVs at high resolution while preserving their native state. By tagging RNA molecules with electron-dense labels or hybridizing them with electron-dense carriers such as lipid-based nanoparticles (LCNPs), cryo-TEM enables the visualization of nucleic acids (NAs) loaded within EVs^[130-132]^. Moreover, fluorescence in situ hybridization (FISH) techniques can be employed to visualize RNA molecules within EVs. The covalent attachment of a fluorophore to a probe specifically designed to hybridize with a target RNA enabled the visualization of mRNA in EV donor and recipient cells *in vitro* using fluorescence microscopy^[133]^. Among advanced FISH techniques, RNA-scope has emerged as a highly sensitive and specific method capable of detecting single RNA molecules with minimal background noise, thanks to its unique hybridization-based signal amplification strategy^[134]^. This method is particularly suited for low-abundance RNA detection and offers potential for application in EV studies. Additionally, multiplexed error-robust FISH (MERFISH) and sequential FISH (seqFISH) significantly expand the capabilities of traditional FISH by enabling the simultaneous detection of hundreds to thousands of distinct RNA species within a single experiment^[135]^.

EV dosing inconsistencies remain another major hurdle. The use of different dose units, or even the absence of dose reporting, poses significant challenges for the reproducibility and comparison of results. In addition, doses are typically reported based on the EV quantity rather than the actual RNA payload, making it difficult to assess the actual therapeutic dose of the sncRNA-EV formulation.

The variability in reported EV dosages likely arises from several factors, including the type of donor cells, EV yield, and the degree and type of internalization by recipient cells. The source of EVs plays a critical role in determining EV yield and composition. Indeed, one of the main challenges of EV-based therapeutics is the scalability of EV production. At least 10-100 μg are typically required to achieve an effective dose response; however, less than 1 μg of EV protein is typically recovered in 1 mL of culture medium^[136]^. A large number of cells and extensive growth surface area are necessary to achieve a good yield, but this may not be feasible for all cell lines due to differences in proliferation rates and associated costs. The observed variability in *in vitro* dosages likely stems from differences in EV yield, which may require adjusting experimental conditions based on available EV quantities.

RNA delivery via EVs is a complex process involving multiple critical steps. First, EVs must be internalized by recipient cells. This uptake occurs through various pathways such as clathrin-mediated endocytosis, caveolin-mediated endocytosis, phagocytosis, micropinocytosis, or direct fusion with the plasma membrane, and not all cell types display the same internalization rate^[137]^. Once inside the cell, the EVs enter endosomes, where they face the challenge of endosomal escape. RNA cargo must escape the degradative pathway within the endosome-lysosome and reach the cytoplasm to exert functional activity^[138]^. However, a substantial fraction of internalized EVs is sequestered and degraded within the endo-lysosomal pathway before their cargo can be released into the cytoplasm, significantly reducing the bioavailable dose^[130,140]^. This intracellular challenge contributes to the difficulty in quantifying the actual functional dose delivered, even when EV quantity is well-defined. Consequently, a standardized effective therapeutic dose in in vitro assays has yet to be defined for sncRNA formulations. Surface engineering with fusogenic proteins has emerged as a promising strategy to enhance the endosomal escape, improving EV uptake and the effective release of their cargo within recipient cells^[141-143]^. Despite great potential, it must be considered that the introduction of viral proteins might induce immunogenic reactions to administered EVs.

This lack of consensus in *in vitro* dose is further reflected in *in vivo* studies, where considerable variability is observed across key experimental parameters, including single versus multiple administrations, disease-specific dosing, and local versus systemic routes of delivery. Regarding administration frequency, the cumulative dose alone does not always determine therapeutic success. Indeed, the variability observed in the cumulative doses reported across studies suggests that the therapeutic outcomes may depend more on the specific disease model, the targeted tissue, or the experimental setup, rather than on the total dose administered. For example, chronic diseases may require lower, repeated doses to maintain therapeutic effects, while acute conditions may benefit from a single, higher dose. An important consideration that could be raised in view of the therapeutic application of EVs is whether repeated administration triggers immune responses or alters pharmacokinetics. A study showed that repeated dosing, rather than high dose itself, led to accelerated clearance. Specifically, repeated administration at higher doses resulted in faster clearance than at lower doses^[144]^.

Variability also emerges when examining dose selection across disease models. Tissues that are difficult to reach, such as the brain, should require higher doses due to the additional challenge of crossing barriers like the blood-brain barrier (BBB). Interestingly, studies targeting Parkinson’s disease used dosages in the order of 10 to 10^2^ μg EV protein, a range totally superimposable to those used for peripheral diseases^[27,48]^. In the context of cancer therapy, the variability in EV dosing is particularly evident. Ohno *et al.* engineered HEK293 cells to express the EGFR-targeting GE11-peptide fused with the transmembrane domain of the platelet-derived growth factor receptor, as a targeting strategy against cancer cells that overexpress EGFR. EVs derived from these modified cells were loaded with miRNA let-7a and systemically administered. Injection of 1 µg of these EVs, once per week for 4 weeks, induced an increase in tumor accumulation and exhibited an antitumor effect mediated by miRNA let-7a^[81]^. Naseri *et al.* intravenously injected (IV) 30 µg MSCs-EVs loaded with LNA-anti-miR-142-3p in breast tumor-bearing mice. Upon administration, *in vivo* results demonstrated a downregulation of miR-142-3p and miR-150, the subsequent upregulation of the associated tumor suppressor genes, including APC and P2X7R, and a significant inhibitory effect on tumor growth rate^[88]^. Systemic administration of 100 μg CRC‐exosomes loaded with functional miRNAs, every 3 days six times, inhibited tumor growth in a mouse model of colorectal carcinoma and increased overall survival^[69]^. Zhang *et al.* demonstrated that intravenous injection of 60 μg miR-665-loaded EVs every 2 days for five weeks inhibited the progression of osteosarcoma *in vivo*^[53]^. Interestingly, a recent study focused on a novel therapeutic approach for treating sarcopenia using a hierarchically injectable hydrogel that sequentially delivers antagomiR-467a-3p, -874-5p-loaded EVs, demonstrated that the intramuscular injection of 200 ng EVs significantly improves sarcopenia in ovariectomized mice^[92]^.

The administration route introduces an additional layer of variability. Systemic administration of EVs often leads to rapid clearance by the mononuclear phagocyte system, with accumulation in organs such as the liver, spleen, lungs, and kidneys within minutes to hours^[145,146]^. The circulation time of EVs is typically short, with reported half-lives of less than 10 min following intravenous injection in mice^[147]^. Given this rapid clearance, one should expect that higher systemic doses might be required to achieve therapeutic effects at the target site. In contrast, local administration enables more targeted delivery and should require lower doses. However, in the studies reviewed, systemic administration did not consistently involve higher doses compared to local routes. This finding may suggest that, in the present studies, the administration route may not significantly impact the total dosage applied. Alternatively, it might reflect, similarly to what was stated before for *in vitro* studies, the experimental need to find an optimal compromise between EV pharmacokinetics and pharmacodynamics, and EV availability.

In summary, while EVs represent a promising and versatile platform for RNA delivery, their clinical translation is still hindered by methodological heterogeneity. A central issue identified in this review is the lack of standardization in EV quantification and dosing strategies. In many studies, EV dosage is either not reported or expressed using different measurement units, making cross-study comparisons difficult. This variability is further amplified by divergent RNA loading protocols and inconsistent dosing regimens in both *in vitro* and *in vivo* models. To address these challenges, the development of protocols that clearly define the RNA-to-EV ratio is essential. Integrating strategies such as RNase treatment combined with qPCR can improve the accuracy of RNA loading assessment. Furthermore, the establishment of standardized *in vitro* potency assays and disease-specific *in vivo* dosing protocols will be critical for enabling the clinical translation of EV-based RNA therapeutics. Despite the existing challenges, significant progress has already been made in the field. Moving forward, sustained advancement will require open collaboration and collective efforts to define benchmark standards, improve reproducibility, and enable reliable cross-study comparisons, ultimately paving the way for successful clinical application of EV-mediated RNA therapies.

## DECLARATIONS

### Availability of data and materials

Data extrapolated from the literature used for the analyses are available from the corresponding authors upon reasonable request.

### Copyright

© The Author(s) 2025.
